# Recent progress in the application of omics technologies in the study of bio-mining microorganisms from extreme environments

**DOI:** 10.1186/s12934-021-01671-7

**Published:** 2021-09-08

**Authors:** Min Li, Jianping Wen

**Affiliations:** 1 Key Laboratory of Systems Bioengineering, Tianjin University, Tianjin, China; 2 Collaborative Innovation Center of Chemical Science and Engineering, Tianjin University, Tianjin, China; 3 Frontier Science Center of Ministry of Education, Tianjin University, Tianjin, China

**Keywords:** Bio-mining microorganisms, Omics technologies, Genomics, Metagenomics, Transcriptomics, Metatranscriptomics, Proteomics, Metaproteomics, Metabolomics

## Abstract

Bio-mining microorganisms are a key factor affecting the metal recovery rate of bio-leaching, which inevitably produces an extremely acidic environment. As a powerful tool for exploring the adaptive mechanisms of microorganisms in extreme environments, omics technologies can greatly aid our understanding of bio-mining microorganisms and their communities on the gene, mRNA, and protein levels. These omics technologies have their own advantages in exploring microbial diversity, adaptive evolution, changes in metabolic characteristics, and resistance mechanisms of single strains or their communities to extreme environments. These technologies can also be used to discover potential new genes, enzymes, metabolites, metabolic pathways, and species. In addition, integrated multi-omics analysis can link information at different biomolecular levels, thereby obtaining more accurate and complete global adaptation mechanisms of bio-mining microorganisms. This review introduces the current status and future trends in the application of omics technologies in the study of bio-mining microorganisms and their communities in extreme environments.

## Introduction

Bio-mining is generally divided into bio-leaching and bio-oxidation of minerals, but the principles of these two processes are basically the same. Bio-leaching refers to the dissolution of metal sulfides (e.g. FeS_2_, CuS_2_ and ZnS) under the action of microorganisms, thereby separating the target metal into the aqueous solution [1]. In contrast, bio-oxidation refers to the microbial breakdown of the mineral matrix surrounding the target metal (usually gold or silver), thereby exposing the target metal to the oxidation slag. During this process, bio-mining activities produce acid mine drainage (AMD). Because of this type of microbial activity, industrial bio-mining and AMD produce extreme environments characterized by low pH and high metal concentrations [2]. Other extreme conditions, such as high salt, high temperature, low temperature and organic solvents, have also been widely reported in bio-mining. At present, the most common and successful microorganisms used in these environments mainly include *Acidithiobacillus ferrooxidans*, *A. thiooxidans*, *A. caluds, Leptospirillum ferrooxidans*, *Sulphobacillus* sp. and *Ferroplasma* sp. [3]. Some acidobacteria, such as *Acidihalobacter prosperus* and *Acidihalobacter ferrooxidans*, may also be potentially used in bio-metallurgy [4]. These bio-mining microorganisms have been industrialized in the recovery of copper, zinc, nickel, cobalt, uranium, gold, silver and other metals through a variety of technologies (such as heap, dump, in-situ and tank reactor bio-leaching) [5].

Traditional molecular techniques that are used to investigate the structure, function and dynamics of microbial communities in bio-mining systems mainly include molecular fingerprinting techniques (such as denaturing gradient gel electrophoresis or end-fragment length polymorphism), fluorescence in situ hybridization, and clone library sequencing [6]. Next-generation sequencing is the most widely used technology for DNA sequencing, based on platforms such as Illumina HiSeq, MiSeq, or NovaSeq 6000. Proteome or metabolome studies are mainly carried out using mass spectrometry combined with chromatographic techniques, such as multi-chromatographic separation and mass spectrometry [7]. The commonly used analytical methods in metabolomics include gas chromatography-mass spectrometry (GC–MS), liquid chromatography-mass spectrometry (LC–MS), nuclear magnetic resonance (NMR) and Fourier transform infrared spectroscopy (FTIR). The application of modern molecular technologies such as genomics, metagenomics, transcriptomics, metatranscriptomics, proteomics, metaproteomics, and metabolomics is deepening our understanding of individual bio-mining microorganisms and microbial communities. These omics techniques can reveal potential functional components and resistance mechanisms of a single bio-mining microorganism and explore its full metabolic potential, thereby constructing highly efficient engineered strains with stronger stress resistance. Such approaches can also explore the complex relationships between members of the bio-mining microbial community, thereby providing a basis for artificially regulating the community to increase its synergistic efficiency. This review summarizes the current status and future trends in the application of omics technologies in the study of bio-mining processes in industrial and natural environments (AMD).

## Application of genomics and metagenomics in bio-mining microorganisms

### Genomics

Genomics refers to the study of the structure and function of all genetic information encoded in an organism's DNA [8]. Genomics can be applied to estimate genetic diversity, study adaptive evolution, discover new genes, establish metabolic models, and predict the response mechanisms of microorganisms to the environment.

At present, the whole genome sequencing results of many bio-mining microorganisms have been uploaded to the NCBI database. Each kind of bio-mining microorganism usually has multiple genotypes, which reflect their genetic diversity. The assembly level of some bio-mining microorganism strains is shown in Table 1, from which it can be seen that the assembly level of the most bio-mining microorganisms is incomplete. Therefore, more complete and comprehensive genomic information needs to be supplemented in the future.

**Table 1:** The assembly level of some bio-mining microbial strains

Organism name	Strains with different assembly levels		
	Complete genome	Scaffold	Contig
*A. ferrooxidans*	YNTRS-40, ATCC 23270, ATCC 53993	YQH-1 [9]	CCM 4253, DSM 16786 [10], Hel18, PQ506 [10], PQ505 [10], CF3 [10], F221, BY0502, RVS1, DLC-5 [11], COP1 [10], S10 [10], BY-3
*A. thiooxidans*	ATCC 19377	CLST	VAN18-5, BN09-1, BC51, DXS-W, ATCC 21835 [10], GD1-3 [12], ATCC 8085 [10], ZBY, DMC [12], A02 [12], Licanantay [13], JYC-17 [12], ATCC 15494 [10], BY-02 [12], A01 [14]
*A. caldus*	MTH-04, ATCC 51756 [15], SM-1 [16]		KU [10], CV18-1 [10], 6 [10], MELC5 [10], BC13 [10], VAN18-3 [10], DX [17], ZBY [17], ZJ [17], MNG [10], C-SH12 [10], F [10], S1 [17]
*L. ferrooxidans*	C2-3 [18]		
*L. ferriphilum*	ML-04 [19], YSK [20]	UBA6657 [21], UBA5695 [21], UBA6673 [21], UBA6674 [21], UBA7872 [21], UBA7391 [21], UBA7384 [21], UBA7870 [21], UBA7392 [21], UBA6677 [21]	DSM 14647 [22], pb_238, DX [23], Sp-Cl [24], ZJ [23], UBA6676 [21], UBA4571 [21], SpSt-902 [25]
*S. thermosulfidooxidans*		CBAR-13, AMDSBA5 [26], DSM 9293, ST, Cutipay [26]	ZJ [27], ZBY [27], DX [27]
*S. thermotolerans*	Kr1 [28]		

Comparative genomics can reveal the adaptive evolution of sequenced bio-mining microorganisms by comparing their genomes. For example, this technology has studied different strains of bio-mining microorganisms such as *A. caldus* [17], *L. ferriphilum* [29], *A. thiooxidans* [12] and *S. thermosulfidooxidans* [27]. These studies emphasized that intraspecific differences and adaptive evolution to extreme niches were related to genetic flow events, such as gene gains, gene losses and genome rearrangement (e.g. gene duplication and horizontal gene transfer). At the same time, these gene turnover events may be revealed by a variety of mobile genetic elements, such as integrative conjugative and mobile elements, insert sequences and genomic islands. In addition, quantifying these genetic elements to determine their contribution to the evolution is an important research content. Quantitative results of HGT affecting the evolution of *Acidiphilium* showed donors that putatively transferred genes to *Acidiphilium* spp., their relative contributions to total HGT events, and the functional proportions of the HGT functions derived from respective donors [30]. However, molecular signals of donors may be erased over time, which is one reason for the inaccurate quantification of genetic elements. Therefore, how to get more accurate quantitative results will be the focus of the future.

Genomics has advantages in discovering new functional genes and metabolic pathways. For example, a novel double cytochrome coding gene cluster (named *tce*) involved in ferrous oxidation was discovered in *A. ferrooxidans* ATCC 23270 for the first time [31]. The typical methyl citrate cycle, which was discovered in the genus *Sulfobacillus*, can exert functions related to energy metabolism and/or resistance in *S. thermotolerans* Kr1 [28]. These new discoveries are useful for inferring the metabolic characteristics of bio-mining microorganisms and perfecting the corresponding metabolic models. The major pathways of carbon and nitrogen metabolism present in some common bio-mining microorganisms are shown in Table 2. In addition, predicted regulatory models for inorganic ion uptake and assimilation and whole-cell model for *A. ferrooxidans* ATCC 23270 have been preliminarily constructed [32]. The models of Fe^2+^ and sulfur oxidation electron transport pathway in *A. ferrooxidans* have characterized some genes and proteins involved in iron sulfur oxidation [33]. Predicted models of carbon assimilation, nitrogen metabolism and sulfur oxidation in *A. thiooxidans* strains have been reported [34]. The acid resistance model in *Leptospirillum* is mainly divided into the first line of defense that prevents the uptake of protons into the cell and second line of defense that neutralizes or expels the protons that enter the cell [35]. However, there are still uncertain factors in the model, such as the unknown function of the genes encoding orphan hypothetical proteins. The interaction models of arsenic with the ars operon related to arsenic resistance in *L. ferriphilum* YSK and *A. thiooxidans* A01 have been shown separately [20]. Notably, these predicted metabolic models are not completely clear, such as unknown genes and their functions. For example, the genes coupling iron-sulfur metabolism have not yet been determined.

**Table 2:** Pathways of carbon and nitrogen metabolism of some common bio-mining microorganisms

Organism	Carbon catabolism				Nitrogen metabolism			Refs
	CBB	EMP	PPP	TCA	N_2_ fixation	Nitrate reduction	Ammonium uptake	
*A. ferrooxidans* ATCC 23270	P	P	P	I	P	A	P	[32]
*A. ferrooxidans* ATCC 53993	P	P	P	I	P	A	P	[36]
*A. ferrooxidans* YQH-1	P	P	P	I	P	A	P	[36]
*A. ferrivorans* SS3	P	P	P	I	P	D/An	P	[37]
*A. ferrivorans* CF27	P	P	P	I	P	D/An	P	[36]
*A. thiooxidans* ATCC 19377	P	P	P	I	A	D/An	P	[36]
*A. thiooxidans* A01	P	P	P	I	A	D/An	P	[38]
*A. thiooxidans* Licanantay	P	P	P	I	A	D/An	P	[13]
*A. caldus* ATCC 51,756	P	P	P	I	A	D/An	P	[15]
*A. caldus* SM-1	P	P	P	I	A	D/An	P	[16]
*A. caldus* DX	P	P	P	I	A	D/An	P	[17]
*A. caldus* ZJ	P	P	P	I	A	D/An	P	[17]
*L. ferriphilum* ML-04	?	I	P	R	P	?	P	[19]
*L. ferriphilum* YSK	?	?	?	?	P	?	?	[20]
*L. ferriphilum* ZJ	A	?	?	I/R	P	D	P	[23]
*L. ferriphilum* DX	A	?	?	I/R	A	D	P	[23]
*L. ferriphilum* DSM 14647	?	?	?	R	A	?	P	[22]
*L. ferrooxidans* C2-3	?	?	?	I/R	P	?	?	[18]
*S. thermotolerans* Kr1	?	?	?	I	?	?	?	[28]
*S. thermosulfidooxidans* DX	P	P	P	P	A	An	P	[27]
*S. thermosulfidooxidans* ZJ	P	P	P	P	A	An	P	[27]

A: absence of the gene/operon or pathway; P: presence of the gene/operon or pathway; I: incomplete pathway; R: reverse tricarboxylic acid cycle; D: dissimilatory nitrate reduction; An: assimilatory nitrate reduction; CBB: Calvin Basham Benson cycle; ?: no information available

Genomics can provide a more comprehensive insight into the response mechanisms of bio-mining microorganisms to environmental conditions. Bio-mining organisms are generally resistant to high concentrations of soluble heavy metals (e.g., arsenic, mercury and silver) and abnormally high concentrations of potentially toxic metals (e.g., copper and iron). Especially, excessive copper and iron can cause oxidative stress in bio-mining microorganisms, affect the intracellular macromolecular activities (such as iron-sulfur metabolism-related enzymes) and reduce the leaching efficiency [39, 40]. However, genome sequences of 44 acidophilic bacteria and archaea lacked genes encoding typical oxidative stress response regulators and exhibited a reduced expression of classic ROS depleting enzymes (e.g. catalase) [40]. Instead, these acidophiles were predicted to possess corresponding alternative systems, such as H_2_O_2_ scavenging rubrerythrin and the Fur family of regulators. Under high salt conditions, resistance mechanisms of *A. thiooxidans* CLST [41] and *L. ferriphilum* Sp-Cl [24] and four members of the *Acidihalobacter* genera [4] have been partially elucidated. Complete genome analysis of twelve iron oxidating bacteria and archaea revealed that the complete or partial genes of the K^+^ transporters Kdp and Ygg were found in most of the genomes [42]. Besides potassium transport, the strategy of these bio-mining microorganisms to adapt to osmotic pressure is mainly the synthesis of compatible solutes, such as betaines, ectoine, glucans, sucrose, glycine, and proline. At low temperature, cold adaptation mechanisms of *A. ferrivorans* XJFY6S-08 mainly included RNA metabolism, molecular chaperones and helicases, membrane modifications, compatible solutes, biofilm and extracellular polysaccharides [43]. These possible mechanisms in extreme environments can be inferred through genomics, but their sequence cannot be completely determined.

### Metagenomics

Metagenomics directly extracts the DNA of all microorganisms from environmental samples to analyze the microbial diversity, population structure, evolutionary relationship, functional activity and the relationship between community and environment. It also has the potential to discover new species, genes, enzymes and compounds.

Metagenomics technology can reconstruct the genomes of microbial diversity communities, which demonstrates its advantages in studying complex biological systems. The first metagenomics study of AMD reported the nearly complete genome reconstruction of *Leptospirillum* group II and *Ferroplasma* type II [44]. Another study also determined the nearly complete genome of the dominant bacteria of the microbial community from a gold mine tailing water pit [45]. However, this technique may yield unsatisfactory results in environments with high species richness, heterogeneities in the abundance of community members, as well as by extensive genome rearrangements.

Metagenomics technology can analyze the structural and functional changes of bio-mining microbial communities in different environments and establish corresponding comprehensive models. The inoculated leaching solution and the leaching heap of a biological heap leaching system have different dominant genera [46]. Specifically, the former was the autotrophic genus *Acidithiobacillus*, and the latter was the heterotrophic genus *Acidiphilium*. This leaching system contained numerous genes related to transposition, DNA repair, and heavy metal transport. The microbial communities on the surface of a copper bio-leaching heap were mainly *Acidithiobacillus*-like, *Thiobacillus*-like and *Leptospirillum*-like microorganisms [47]. They are major participants in key metabolic pathways (carbon fixation, nitrogen metabolism, ferrous iron oxidation and sulfur metabolism). High-resolution targeted metagenomics technology has been used to analyze the changes in the community structure during adaptation to extreme acidity and high concentrations of gold concentrate pulp for a long time [48]. The genomes of the dominant strains in this community (*A. ferrooxidans* etc.) were enriched with functional genes related to heavy metal transport and stress resistance. Similarly, analysis of AMD microbial communities at low temperatures (6–10 °C) showed that *A. ferrivorans*-like strains were the most abundant microorganisms [49]. To a wide range of hot spring temperatures, the adaptation of *Acidithiobacillus* populations to high temperatures was mainly related to their high GC and proline encoding contents [50].

Metagenomics techniques can provide insights into microbial community members with relatively low abundance and lead to the discovery of new species. These newly discovered biological resources are likely to be uncultured microorganisms that cannot be cultured in the laboratory at present. For example, a new uncultured psychrotolerant iron- and sulfur-oxidizing acidophile from the family Gallionellaceae was identified in the microbial community of the Sherlovaya polymetallic mine [51]. Metagenomic technology can also realize the rapid screening of target genes and the identification of new genes or gene clusters in uncultured bacteria. For example, metagenomic microarrays have been used for the rapid screening of genomic fragments containing RuBisCO type I large subunit genes (*cbbL*) and novel *nif* gene clusters [52, 53].

In summary, since complete genome sequences are available for only a few bio-mining microorganisms, more complete genome data need to be supplemented in the future. At the same time, the lack of these data also limits the elucidation of the metabolic mechanisms of bio-mining microorganisms to a certain extent. Genome analysis of the above bio-mining microorganisms only allows the prediction of genetic and metabolic potential, while transcriptomic, proteomic, and metabolomic information are still needed to study the expression of the identified candidate genes, as well as its temporal and spatial changes.

## Application of transcriptomics and metatranscriptomics in the study of bio-mining microorganisms

### Transcriptomics

Transcriptomics refers to the study of RNA molecules transcribed by an organism using high-throughput methods, such as microarray and RNA-seq [8]. Transcriptomics can explore the dynamic changes in gene expression of bio-mining microorganisms caused by changes in different environments and specific genes.

DNA microarray is a technology originally used for the transcriptomic study of various bio-mining microorganisms. This technology can elucidate the regulatory strategies of bio-mining microorganisms exposed to organic extractants, quorum sensing super-agonists, extreme pH and other stress conditions. When exposed to an organic extractant (Lix984n), the expression of genes related to the pentose phosphate pathway, fatty acid and glutamate biosynthesis in *A. ferrooxidans* ATCC 23270 was upregulated [54]. The specific strategy through which *A. ferrooxidans* deals with short-term stress was to continuously enhance the expression of genes encoding proteins involved in electron transport, such as *petI*, *petII*, *cyo* and *cyd*. Under the stimulation of a synthetic super-agonist AHL analogue, the transcriptome of *A. ferrooxidans* indicated that quorum sensing regulates the *afeI* gene encoding AHL synthase rather than the gene encoding the quorum sensing transcription regulator AfeR [55]. Under stress due to increased pH, *A. ferrooxidans* can promote the pH homeostasis of cytoplasm through hydrogen absorption and nitrogen fixation [56].

Compared with DNA microarray technology, RNA-seq has higher sensitivity, accuracy and resolution, so it is increasingly used in transcriptomic studies [57]. This technology has been widely used to understand the adaptive mechanisms of bio-mining microorganisms in different environments, such as different energy substrates, pH and heavy metal ions. The RISCs oxidation model and genes involved in ferrous oxidation can be initially revealed by comparing the gene expression profile differences of *A. ferriphilus* SCUT-1 in Fe^2+^, S^0^ and FeS_2_ media [58]. Similarly, differentially expressed genes with S^0^ and S_2_O_3_^2−^ as energy substrates played a key role in revealing new insights into the sulfur metabolism model of *A. thiooxidans* ATCC 19377 [59]. The differentially expressed genes of *A. thioxidans* CCTCC M 2012104 [60] and *A. caldus* CCTCC M 2018054 [61] under different acidity systems were studied separately. The results indicated they had similar potential antacid components and resistance mechanisms, such as the changes in genes related to unsaturated fatty acids, sulfur metabolism, carbon metabolism and flagella assembly. Under the low copper and higher copper stress, cusRS encoding Cu^2+^ efflux system was considered being an important gene involved in the copper resistance mechanism of *A. caldus* CCTCC M 2018727 [39]. Under Mg^2+^ stress, ssRNA-seq results showed that the expression of genes related to type IV fimbriae and carbon fixation in *A. ferrooxidans* decreased [62]. In addition, RNA-seq technology can also study engineering strains to understand the impact of specific proteins on overall metabolism. For example, in order to understand the function of the ferric uptake regulator (Fur) in resisting extreme acidic environments, the overall transcriptome changes of the wild-type *A. caldus* MTH-04 strain and its Fur knockout strain were compared [63].

### Metatranscriptomics

Metatranscriptomics is based on high-throughput sequencing of total RNA or mRNA to study the gene expression and regulation of microbial communities from environmental samples. It can evaluate the community structure and function changes of bio-mining microorganisms in different time and space, getting active expression profiles and community activities in response to changing environments. For example, the environmental transcriptome provided evidence for the metabolic differences between biofilms and planktonic cells of *Leptospirillum* spp. in its natural microbial community [64]. Specifically, the metabolism of attached cells was dominated by mixed acid fermentation, while the TCA cycle of planktonic cells was more active. In addition, the transcriptome comparison of biofilms grown in the natural environment and in a laboratory bioreactor revealed that the genus *Leptospirillum* was dominant in all samples [65]. However, there are significant differences in the number and types of low-abundance members in these two samples. This study also emphasized that environmental factors, rather than the developmental stage of biofilms, were important in driving biofilm diversity.

Metatranscriptomics can also help clarify the biological roles played by community members in the bio-mining process. For example, the differentially expressed genes in the early and late stages of a coculture community composed of six strains were analyzed. In the early stage, the expression of genes involved in cellular components, genes involved in binding in *S. thermosulfidooxidans* and genes involved in catalytic activities in *A. caldus* were up-regulated, indicating that the microorganisms maintain their physiological activities by promoting cell proliferation [66]. In the latter stage, genes related to signal transduction and stress response in *L. ferriphilum*, genes related to establishing localization in *A. caldus*, and transporter genes in *S. thermosulfidooxidans* were highly expressed to resist the severe environmental stress caused by the accumulation of metal ions, toxins and metabolites [66]. However, metatranscriptomics cannot clearly clarify the complete gene expression and metabolic changes of each member. This is because of the complexity of biological systems, such as the overlapping parts of the members' genomes.

In summary, transcriptomics and metatranscriptomics are less commonly used in the study of bio-mining microorganisms than other omics approaches, which may be because of the limitation of the short sequence reads of the associated techniques. Specifically, these reads cannot always achieve a true reconstruction of the original sequence, faithful analysis of the complete sequence of transcripts with multiple isotypes, and direct sequencing of RNA modifications. Long-read transcriptomics and CITE-seq/spatial transcriptomics are rarely applied to bio-mining microorganisms, but this approach may be a major future trend.

## Application of proteomics and metaproteomics in the study of bio-mining microorganisms

### Proteomics

Similar to transcriptomics, proteomics can perform qualitative and quantitative analyses of all proteins expressed by bio-mining microorganisms under different stress conditions. Currently, comparative proteomics is the most widely used in bio-mining microorganisms. The obtained differentially expressed proteins valuable for inferring the response of strains to environmental disturbances. For example, proteomics was applied to study the stress response and physiological adaptation of *A. ferrooxidans* to pH [67], phosphate starvation [68, 69], temperature [70], different energy substrates [71–73], and different lifestyles (attached and planktonic cells) [74, 75]. Proteomic changes in *A. ferrooxidans* in response to heavy metals and potentially toxic micronutrients have also been reported, including copper [76–79], cadmium [80], uranium [81], and potassium [82]. To further determine the interactions between the peripheral components of bio-mining microorganisms and the oxidizable substrates, a proteomics study was conducted on the periplasmic space. The first proteomic analysis of the periplasmic space, the primary site for oxidation of iron and RISCs in *A. ferrooxidans*, identified 131 proteins [83]. Notably, quantitative proteomics is essential for a comprehensive understanding of adaptive biochemical mechanisms within strains. The proteomic analysis of *A. ferrooxidans* ATCC 53993 studied by isotope-coded protein labeling at high concentrations of copper revealed the high overexpression of proteins encoded by several genes in a unique genomic island [79].

Proteomics can identify the changes of intracellular function and metabolic pathway by identifying the changes of proteins. When studying the proteomic response of *A. caldus* to low pH, it was found that the enzymes involved in sulfur metabolism and PspA were up-regulated, while glutamate decarboxylase was uniquely expressed [84]. The response of *A. caldus* SM-1 to NaCl stress was found to involve the upregulation of several heat shock proteins, enzymes involved in proline biosynthesis, two DNA-binding proteins and another single-stranded DNA binding protein [85]. The cellular response of *L. ferriphilum* ML-04 to arsenic stress involved specific arsenic-resistance proteins, as well as proteins involved in phosphate metabolism, protection from reactive oxygen species, glutathione metabolism, DNA synthesis and repair, as well as protein synthesis, folding and refolding [86]. By analyzing the differentially expressed proteins of *S. thermotolerans* cells exposed to high arsenic gold-containing sulfide concentrates, it was found that stress-resistance proteins such as MBL folding metallohydrolase, quinone oxidoreductase and GroEL chaperones were critical in resisting arsenic and sulfide stress [87]. These studies sometimes discuss some proteins with unknown functions. For example, a study discussed a hypothetical new function of Hdr in bio-mining microorganisms, that is, its three subunits are involved in S^0^ oxidation [88].

### Metaproteomics

Metaproteomics studies the entire protein pool directly recovered from complex environmental microbial communities at a given point in time [89]. Metaproteomics can infer genomic information, metabolic potential, microbial diversity, and niche distribution by identifying active proteins. The bio-mining microbial communities studied by metaproteomics technology to date mainly come from AMD. The first proteomics study of an AMD microbial biofilm community was published in 2005, and it mainly examined the division of metabolic functions in the community [90]. Proteomic data can be used to assess the presence of genome types and activities [91]. For example, genomic typing inferred by proteomics identified recombinants and active (sub)populations within the environment [92]. Moreover, metaproteomics combined with isotope labeling techniques can also be used to understand the nutrient flow patterns of microbial community members and metabolic responses in mixed microbial systems. For example, the differential fractionation of stable hydrogen isotopes in proteins can reveal the nutrient levels of microbial community members because of their species specificity [93]. Using ^2^H and ^15^N isotopes in a proteomic SIP experiment showed that archaea in AMD biofilms obtain nitrogen by recycling nitrogen-containing biomolecules [94].

When proteomics was used to study the ecological distribution and population physiology, it was found that the strong correlation between physiological state and community members exceeded any correlation with single environmental factors or their combinations [95]. Based on the quantification of the relative protein abundance in the late stage of biofilm growth, a study inferred the physiological changes caused by the competitive interaction of community members [96]. The effects of environmental factors such as pH and temperature on community proteome have also been studied [97, 98]. In addition, the protein abundances in the laboratory and the natural environment were quantitatively compared to detect the functional level and metabolic response of the mixed microbial system [99]. This study emphasized that an increased abundance of stress response proteins in laboratory communities caused their growth rate to be slower than that of natural communities. Notably, structural proteomics studied ecological adaptation and microbial evolution by characterizing the prevalence and dynamics of protein post-translational modifications (PTMs) [100]. In particular, the detection of PTM on Cas proteins involved in antiviral defense was a breakthrough. Besides AMD microbial communities, proteomic changes of mixed cultures of *A. ferroxidans* and *A. caldus* under chloride stress during pyrite bio-leaching have been characterized. The results showed acidophilic microorganisms adapt to NaCl stress mainly by modifying their cell membrane, accumulating amino acids that may be used as osmotic protectants, and expressing YceI family proteins involved in resistance to acid and osmotic stress [101].

In summary, there are still many proteins that cannot be identified through proteomics, which is caused by the limited accuracy and sensitivity of existing mass spectrometry techniques. Soon, mass spectrometry technology that cannot tell the entire protein sequence may be replaced by potential new technologies, such as fluorescent protein fingerprinting, nanopore 5D fingerprinting and sub-nanopore arrays. This indicates that future research focus of proteomics and metaproteomics is still the discovery and functional characterization of new proteins.

## Application of metabolomics in the study of bio-mining microorganisms

Untargeted metabonomics is a global analysis of all similar types of molecules, while targeted metabonomics quantifies a suite of identified molecules [8]. The first untargeted and targeted metabolomics study on *A. ferrooxidans* Wenelen and *A. thiooxidans* Licanantay was published in 2013 [102]. This study examined the differences in the metabolic profiles of attached and planktonic cells with different energy sources, and highlighted key active pathways. Specifically, the active metabolic pathways in the two strains included the synthesis of glutathione, polyamine, and amino acids and metabolites related to energy processes. Especially, spermidine was detected in both extracellular and intracellular samples of these two strains, implying its potential as a biomarker of sulfur oxidation activity in bio-leaching processes.

In summary, metabolomics was less frequently applied in research on bio-mining microorganisms, which may be limited by the complexity of the metabolome composed of many biomolecules with different physical and chemical properties. Technical limitations have also resulted in fewer metabolomic studies in these organisms. Specifically, there is still no single analytical method that can fully cover all the metabolic components of an environmental sample. Although the combination of multiple analytical methods (such as GC–MS, LC–MS, and NMR) has eased this problem to a certain extent, it is necessary to develop more advanced technologies to identify all metabolites of bio-mining microorganisms in the future.

## Application of multi-omics in the study of bio-mining microorganisms

There is increasing evidence that integrating multi-omics data is a more powerful approach than single omics analysis, and integrated multi-omics analysis can comprehensively study more complex biological systems and solve more complex problems. Multi-omics can provide more details on the metabolic properties and adaptive mechanisms of individual organisms. Genomics, transcriptomics and proteomics revealed the adaptative strategy of *L. ferriphilum* growing on chalcopyrite, especially identified a previously undiscovered nitrogenase cluster for nitrogen fixation from the atmosphere [103]. Genomics and transcriptome analysis revealed the potential strategies of *A. ferrivorans* YL15 in extremely acidic pH, high metal ion concentrations, UVR, low temperature and intrusion of extraneous genetic elements [104]. Transcriptomic and proteomic provided the first comprehensive insights into the aerobic and anaerobic metabolism of hydrogen by *A. ferrooxidans* at low pH [105]. Transcriptomics can detect information that proteomics may miss, and proteomics can also provide unique information that transcriptomics cannot provide.

Multi-omics can simultaneously reveal the taxonomic composition and functional division of the community and explore their metabolic interactions. For example, the analysis of genomic and proteomic data of 27 communities showed that the differential regulation of shared genes and the influence of the unique genes of each genotype on niche division [106]. Metagenomics and metaproteomics identified the major functions of seven dominant bacteria in the cycle of inorganic (arsenic, iron, sulfur) and organic resources, and established their metabolic interaction model [107]. Similarly, metagenomic and metatranscriptomic revealed the specific gene transcription behaviors of the most active organisms that are related to responses and adaptation to different environmental conditions [108]. In another study, metagenomics and metatranscriptomics emphasized the key role of rare group activities (e.g. nitrogen fixation and sulfur oxidation) in the overall function and assembly of AMD community [109]. Community genomics, transcriptomics and proteomics discovered a new species “Leptospirillum group IV UBA BS” and hypothesized its cooperative interaction with *L. ferrodiazotrophum* in hydrogen metabolism [110]. However, the information provided by multi-omics still cannot realize the detection of all low-abundance members and the elucidation of community member interactions.

In conclusion, although each omics method provides valuable information for the study of bio-mining microorganisms, individual approaches cannot completely reflect the diversity and metabolic versatility of bio-mining microorganisms. Integrating multiple omics technologies can provide more comprehensive microbiome information and richer information for predictive modeling of community phenotypes, enriching existing databases [111]. However, the underlying heterogeneity of individual omics data, the enormous amount of data, and the lack of analysis techniques bring difficulties to the integrated analysis of multi-omics. Therefore, focusing on the efficient integration and de-redundancy of multi-omics data is the focus of future research.

## Conclusions

Omics technology has provided a deep understanding of the response of bio-mining microorganisms to extreme environments. Genomics, transcriptomics, proteomics and metabolomics have their respective contributions to the establishment of metabolic models of individual bio-mining microorganisms. They can also speculate on the evolutionary relationship of strains, discover new substances, and study the interactions between genes and genes, and proteins and proteins. Similarly, metagenomics, metatranscriptomes and metaproteomics can establish comprehensive metabolic models to characterize the interaction of community members and the impact of the external environment on their activities. These techniques can also find low-abundance members and emphasize their important functions. However, individual omics technologies have some shortcomings, such as the limitation of metabolic potential showed by genomics, the poor reproducibility of transcriptomics and proteomics, and the difficulty of identification of metabolism. Although multi-omics can provide more complete and accurate details, it still has much room for progress in analyzing the actual activities of communities in the ever-changing extreme environment. Therefore, if the problem of effectively linking DNA sequencing, RNA sequencing, and mass spectrometry data is overcome, integrated multi-omics analysis will surely enable an unprecedented understanding of bio-mining microorganisms. Last but not least, the continued exploration of resistance mechanisms and the application of omics information to the construction of highly efficient engineering strains are the future development direction.

## Data Availability

Not applicable.

## Abbreviations

AMD: Acid mine drainage
HGT: Horizontal gene transfer
RISCs: Reduced inorganic sulfur compounds
